# Enhancement of vitamin B_6_ levels in rice expressing Arabidopsis vitamin B_6_ biosynthesis *de novo* genes

**DOI:** 10.1111/tpj.14379

**Published:** 2019-07-11

**Authors:** Nathalie Mangel, Jared B. Fudge, Kuan‐Te Li, Ting‐Ying Wu, Takayuki Tohge, Alisdair R. Fernie, Boris Szurek, Teresa B. Fitzpatrick, Wilhelm Gruissem, Hervé Vanderschuren

**Affiliations:** ^1^ Plant Biotechnology, Department of Biology ETH Zürich Zürich Switzerland; ^2^ Department of Botany and Plant Biology University of Geneva Geneva 1211 Switzerland; ^3^ Max‐Planck‐Institute for Molecular Plant Physiology Potsdam‐Gölm 14476 Germany; ^4^ IRD Cirad University of Montpellier IPME Montpellier 34394 France; ^5^ Advanced Plant Biotechnology Center National Chung Hsing University Taichung City 40227 Taiwan; ^6^ Plant Genetics Lab TERRA Research and Teaching Centre Gembloux Agro BioTech University of Liège Gembloux 5030 Belgium; ^7^ Present address: Graduate School of Biological Sciences Nara Institute of Science and Technology Ikoma Nara 630‐0192 Japan

**Keywords:** PDX proteins, monocot, rice, crop, vitamin B_6_, stress

## Abstract

Vitamin B_6_ (pyridoxine) is vital for key metabolic reactions and reported to have antioxidant properties *in planta*. Therefore, enhancement of vitamin B_6_ content has been hypothesized to be a route to improve resistance to biotic and abiotic stresses. Most of the current studies on vitamin B_6_ in plants are on eudicot species, with monocots remaining largely unexplored. In this study, we investigated vitamin B_6_ biosynthesis in rice, with a view to examining the feasibility and impact of enhancing vitamin B_6_ levels. Constitutive expression in rice of two *Arabidopsis thaliana* genes from the vitamin B_6_ biosynthesis *de novo* pathway, At*PDX1.1* and At*PDX2*, resulted in a considerable increase in vitamin B_6_ in leaves (up to 28.3‐fold) and roots (up to 12‐fold), with minimal impact on general growth. Rice lines accumulating high levels of vitamin B_6_ did not display enhanced tolerance to abiotic stress (salt) or biotic stress (resistance to *Xanthomonas oryzae* infection). While a significant increase in vitamin B_6_ content could also be achieved in rice seeds (up to 3.1‐fold), the increase was largely due to its accumulation in seed coat and embryo tissues, with little enhancement observed in the endosperm. However, seed yield was affected in some vitamin B_6_‐enhanced lines. Notably, expression of the transgenes did not affect the expression of the endogenous rice *PDX* genes. Intriguingly, despite transgene expression in leaves and seeds, the corresponding proteins were only detectable in leaves and could not be observed in seeds, possibly pointing to a mode of regulation in this organ.

## Significance Statement

We demonstrate that vitamin B_6_ can be enhanced in a monocot species (rice), leading to increased accumulation of unphosphorylated B_6_ vitamers and pyridoxine glucosides, particularly in leaves. Our analysis reveals current limitations and possibly regulatory mechanisms for vitamin B_6_ biosynthesis in rice endosperm.

## Introduction

Vitamin B_6_ is essential for all kingdoms of life and is composed of a mixture of six different vitamers: pyridoxal (PL), pyridoxine (PN), pyridoxamine (PM), and its 5′‐phosphorylated derivatives: pyridoxal 5′‐phosphate (PLP), pyridoxine 5′‐phosphate (PNP), and pyridoxamine 5′‐phosphate (PMP) (Fitzpatrick *et al*., 2007; Fitzpatrick, 2011). Vitamin B_6_ in its form as PLP is required as a coenzyme for more than 200 enzymatic reactions in cells, primarily in amino acid, sugar, and fatty acid metabolism (Percudani and Peracchi, 2003; Colinas *et al*., 2016). Mammals lack the ability to biosynthesize vitamin B_6_ *de novo*, and plants represent one of their major sources of the vitamin (Fitzpatrick, 2011). Acute vitamin B_6_ deficiency can lead to various chronic diseases in humans, including neurological disorders, cardiovascular disease, and diabetes (Hellmann and Mooney, 2010; Di Salvo *et al*., 2012; Fudge *et al*., 2017).

**Figure 1:**
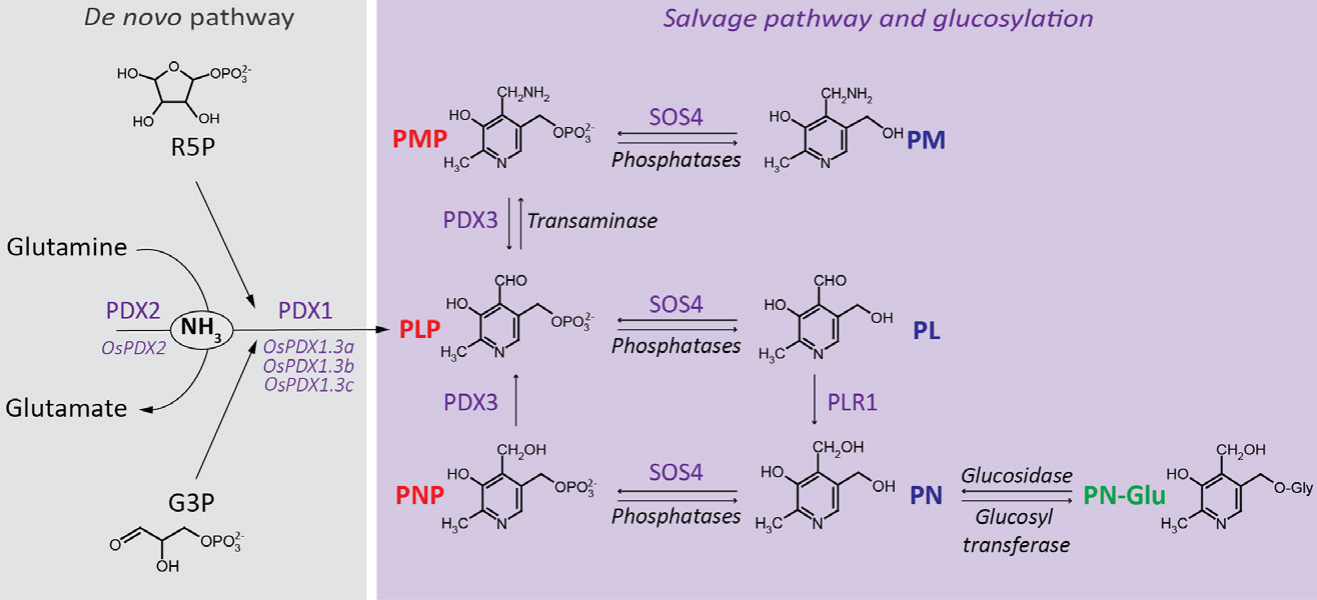
Scheme of the vitamin B_6_ biosynthesis pathway as described in Arabidopsis and extrapolated to rice. Vitamin B_6_ is biosynthesized *de novo* in the presence of R5P (ribose 5‐phosphate), G3P (glyceraldehyde 3‐phosphate) and glutamine through the combined action of PDX1 (encoded by Os*PDX1*.*3a*: LOC_Os07g01020; Os*PDX1.3b*: LOC_Os10g01080; Os*PDX1.3c:* LOC_Os11g48080 in rice) and PDX2 (encoded by Os*PDX2*: LOC_Os02g03740 in rice) to form PLP (pyridoxal 5′‐phosphate). Enzymes from the salvage pathway allow interconversion between the B_6_ vitamers. Annotation in red depicts the phosphorylated B_6_ vitamers: PMP (pyridoxamine 5′‐phosphate), PLP and PNP (pyridoxine 5′‐phosphate); annotation in blue depicts the unphosphorylated B_6_ vitamers: PM (pyridoxamine), PL (pyridoxal) and PN (pyridoxine); annotation in green depicts the glucosylated PN vitamer (PN‐Glu). PDX3 refers to a PN/PM oxidase, SOS4 to a PN/PL/PM kinase and PLR1 to a PL reductase.

In plants, PLP is biosynthesized *de novo* via the so‐called ‘DXP independent pathway’ first characterized in *Arabidopsis thaliana* (Tambasco‐Studart *et al*., 2005; Fitzpatrick *et al*., 2007). PLP biosynthesis *de novo* requires two enzymes, PDX1 and PDX2 (Figure 1), which together act as a glutamine amidotransferase utilizing ribose 5‐phosphate (R5P), glyceraldehyde 3‐phosphate (G3P) and glutamine as substrates (Burns *et al*., 2005; Raschle *et al*., 2005, 2007, 2009; Tambasco‐Studart *et al*., 2005; Moccand *et al*., 2011). In Arabidopsis, there are three PDX1 homologs, only two of which are catalytically active (PDX1.1 and PDX1.3), and only one gene encoding PDX2 (Tambasco‐Studart *et al*., 2005). PLP can also be produced by interconversion of the different B_6_ vitamers through salvage pathways, which are present in all kingdoms of life (Ruiz *et al*., 2008; Colinas and Fitzpatrick, 2015). Among the known enzymes involved in this pathway, a PMP/PNP/PLP kinase named SALT OVERLY SENSITIVE 4 (SOS4) (Lum *et al*., 2002; Shi and Zhu, 2002; Shi *et al*., 2002; Gonzalez *et al*., 2007b), a PMP/PNP oxidase named PDX3 (Gonzalez *et al*., 2007b; Sang *et al*., 2007, 2011; Colinas *et al*., 2016), and a pyridoxal reductase named PLR1 (Herrero *et al*., 2011) have been identified in plants (Figure 1). In addition, plants can convert PN to glucosidic derivatives (pyridoxine glucosides, PN‐Glu), for example, pyridoxine‐5′‐β‐d‐glucoside (PNG), using glucosyltransferases (Gregory and Ink, 1987; Gregory, 1998; Ollilainen, 1999).

B_6_ vitamers are reported to display antioxidant properties (Bilski *et al*., 2000; Danon *et al*., 2005; Denslow *et al*., 2005) and therefore vitamin B_6_ has been implicated in abiotic and biotic stress responses in plants (Fitzpatrick, 2011; Hanson *et al*., 2016). This notion is also supported by the association and susceptibility of Arabidopsis mutants impaired in vitamin B_6_ biosynthesis *de novo* or salvage pathways to various abiotic stresses, including osmotic stress (Chen and Xiong, 2005; Titiz *et al*., 2006), salt stress (Shi *et al*., 2002; Titiz *et al*., 2006), oxidative stress (Chen and Xiong, 2005), heat stress (Moccand *et al*., 2014; Dell'Aglio *et al*., 2017), as well as high light and photo‐oxidative stress (Titiz *et al*., 2006; Havaux *et al*., 2009). Independent studies have also demonstrated that abiotic stresses can modulate the expression of genes of vitamin B_6_ biosynthesis *de novo* and salvage pathways (Shi *et al*., 2002; Savenstrand *et al*., 2004; Denslow *et al*., 2005, 2007; Ristila *et al*., 2011). Coincidently, abiotic stresses also appear to alter vitamin B_6_ levels in Arabidopsis, with a significant increase in PLP and some changes in PL and PN having been reported (Huang *et al*., 2013; Moccand *et al*., 2014). Vitamin B_6_ is also assumed to play a role in plant responses to pathogens, as evidenced by the enhanced sensitivity of Arabidopsis *pdx1.2*,* pdx1.3* and *sos4‐1* mutants to *Pseudomonas syringae* pv. *tomato* DC3000 and *Botrytis cinerea* (Zhang *et al*., 2015). Furthermore, gene expression analyses showed that Arabidopsis *pdx3* mutants display a strong upregulation of genes related to plant defense (Colinas and Fitzpatrick, 2016; Colinas *et al*., 2016). In addition, increased severity of *Botrytis cinerea* symptoms have been observed in tomato, in which expression of *PDX1.2* and *PDX1.3* have been knocked‐down (Zhang *et al*., 2014).

Various reports have demonstrated that vitamin B_6_ content can be increased *in planta* by genetic engineering strategies (Herrero and Daub, 2007; Chen and Xiong, 2009; Raschke *et al*., 2011; Li *et al*., 2015). The combined expression of Arabidopsis *PDX1.1* and *PDX2* has led to a significant increase in vitamin B_6_ content in transgenic Arabidopsis leaves and seeds, as well as field‐grown transgenic cassava leaves and roots (Raschke *et al*., 2011; Li *et al*., 2015). Recent reports have indicated that constitutive expression of Arabidopsis *PDX2* in potato also leads to a significant increase in vitamin B_6_ content (Bagri *et al*., 2018). In Arabidopsis, overexpression of *PDX1.1* and *PDX2* was reported to have pleiotropic effects, including increased size of leaves and embryos (Raschke *et al*., 2011). Overaccumulation of vitamin B_6_ in transgenic Arabidopsis and potato was also associated with increased tolerance to oxidative stress, supporting the proposed antioxidant function of vitamin B_6_ *in planta* (Raschke *et al*., 2011; Bagri *et al*., 2018).

To date, studies investigating vitamin B_6_ biosynthesis in monocot species have been scarce (Yang *et al*., 2017). Within this context, it is interesting to note that recent studies have highlighted the almost exclusive presence of the non‐catalytic PDX1 homolog, PDX1.2, to eudicots (Moccand *et al*., 2014; Dell'Aglio *et al*., 2017). The absence of *PDX1.2* from monocots sets precedence for unraveling further regulatory differences between these two groups of flowering plants. Rice is an important cereal crop for almost half of the global population and its yield is affected by several abiotic and biotic stresses (Seck *et al*., 2012; Zhao *et al*., 2016). The impact of vitamin B_6_ on environmental stress responses in rice has not been studied to date. Furthermore, while rice represents 35–70% of daily caloric intake in several populations (Kennedy *et al*., 2003; Meng *et al*., 2005; Bhullar and Gruissem, 2013), most of the micronutrients, which are primarily stored in the husk, aleurone and embryo, are removed during processing (Kennedy *et al*., 2003; Lucca *et al*., 2006; Bhullar and Gruissem, 2013). Therefore, these populations suffer from deficiencies in one or several essential micronutrients including vitamin B_6_ (Thompson and Amoroso, 2011; Vanderschuren *et al*., 2013; von Grebmer *et al*., 2014; Singh *et al*., 2016; Strobbe and Van Der Straeten, 2018). Thus, enhancing vitamin B_6_ content in rice can provide insight into its impact on rice growth and yield, as well as its tolerance to stress responses, while also providing knowledge on its potential to improve the nutritive value of the consumed grain.

Here, we have studied the effect of heterologous expression of the Arabidopsis *PDX1.1* and *PDX2* transgenes in rice. We performed a phenotypic assessment as well as stress resistance assays to characterize selected transgenic rice lines. We further characterized the selected transgenic rice lines by profiling the expression of their transgenes as well as the identified rice *PDX* genes in leaves, unpolished, and polished seeds. We used a high‐performance liquid chromatography (HPLC)‐based method to report an in‐depth profiling of B_6_ vitamers in wild‐type and transgenic rice lines.

## Results

### Enhancement of vitamin B_6_ content in rice using the Arabidopsis *de novo* pathway

**Figure 2:**
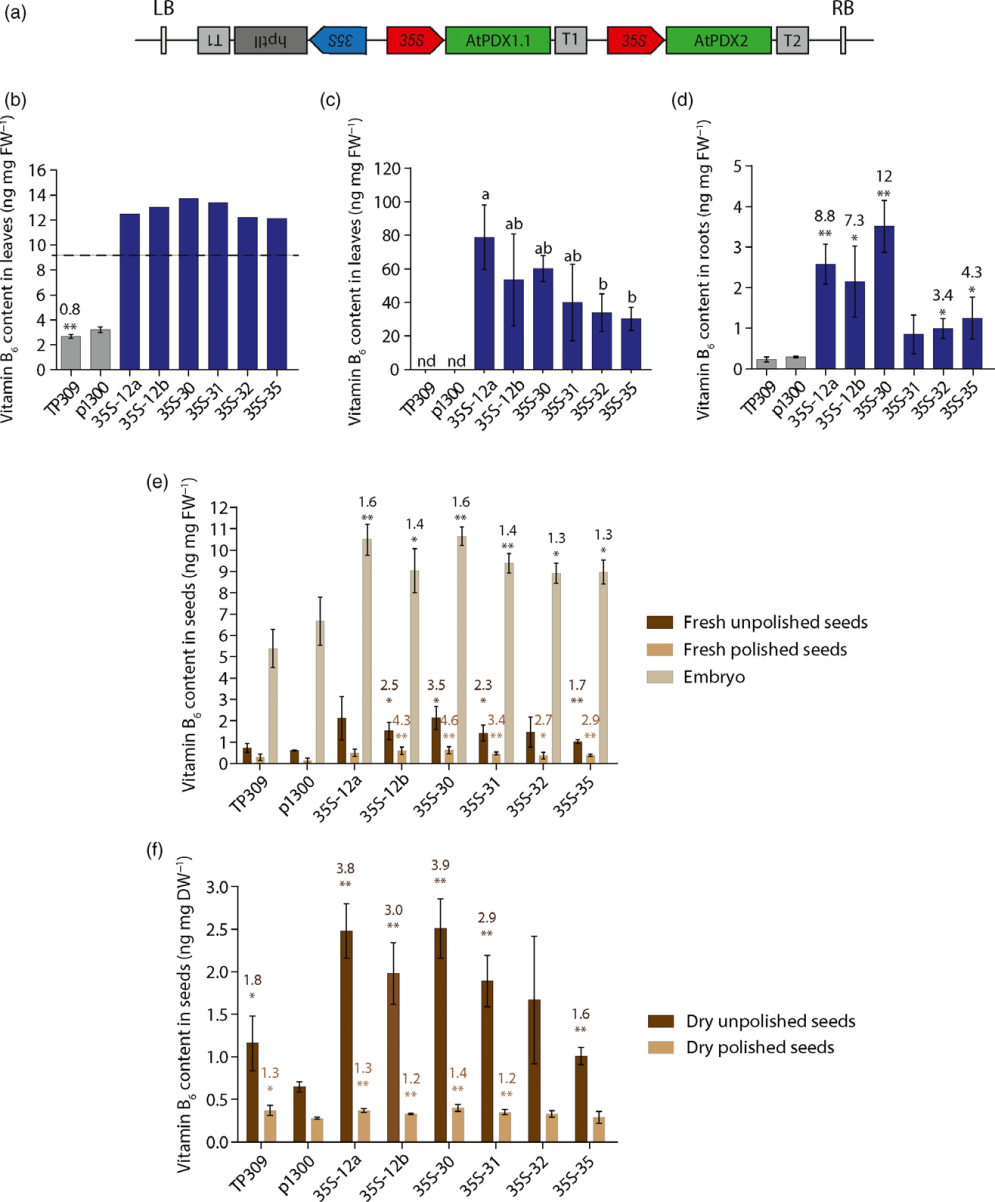
Enhancement of vitamin B_6_ content in rice by heterologous expression of Arabidopsis PDX1/PDX2. (a) Schematic representation of the T‐DNA regions of the transformation vector used to generate the *35S* lines. LB, Left Border; RB, Right Border; *35S*, cauliflower mosaic virus *35S* (*CaMV 35S*) promoter; At*PDX1.1*, At2g38230 coding sequence; At*PDX2*, At5g60540 coding sequence; *hptII*,* hygromycin* phosphotransferase gene; T1, *CaMV 35S* terminator; T2, Octopine synthase terminator. (b) Vitamin B_6_ content in leaf sample extracts of *35S* lines in the T_2_ generation compared to wild‐type (TP309) and empty vector control (p1300). Average ± SD of four biological replicates. Student's *t‐*test (p1300 versus transgenic lines and TP309), *P < 0.05, **P < 0.01. Standard deviation is not indicated for samples with at least one O.D. (Optical Density) value above the linear range of the standard curve indicated by the area above the dotted line. (c) Vitamin B_6_ content as in (b) in 15‐fold diluted leaf sample extracts. Average ± SD of four biological replicates. Tukey's multiple comparison test (*P *< 0.05). nd, not detected. (d) Vitamin B_6_ content in root sample extracts from mature plants in the T_2_ generation grown under greenhouse conditions. Average ± SD of four biological replicates. Student's *t*‐test (p1300 versus transgenic lines and TP309), *P < 0.05, **P < 0.01. Values above the bars represent the fold increase compared to p1300. (e) Vitamin B_6_ content in fresh mature unpolished and polished seeds and in the embryo from plants in the T_2_ generation grown in the greenhouse. Average ± SD of four biological replicates. Student's *t*‐test (p1300 versus transgenic lines and TP309), *P < 0.05, **P < 0.01. (f) Vitamin B_6_ content in dry mature unpolished and polished seeds of the samples as in (e). Average ± SD of four biological replicates. Student's *t*‐test (p1300 versus transgenic lines and TP309), *P < 0.05, **P < 0.01. Values above the bars represent the fold increase compared to p1300. For panels (b) to (f), the vitamin B_6_ content was determined by a yeast bioassay.

In order to enhance the vitamin B_6_ content in rice, we transformed rice variety Taipei 309 (TP309) with binary vectors carrying both the Arabidopsis *PDX1.1* and *PDX2* genes under the constitutive *CaMV 35S* promoter (*p35S::*At*PDX1.1‐p35S::*At*PDX2*) (Figure 2a). We selected Arabidopsis genes because they were previously demonstrated to enhance vitamin B_6_ content in cassava (Li *et al*., 2015). The use of Arabidopsis gene sequences also aimed at avoiding unintentional silencing of endogenous *PDX* rice genes. A molecular characterization of the transgenic rice lines was performed at the T_0_ generation assessing 26 lines for the presence of the *p35S::*At*PDX1.1‐p35S::*At*PDX2* construct (hereafter called *35S* lines). Polymerase chain reaction (PCR) genotyping indicated that 21 of the *35S* lines harbored both of the Arabidopsis *PDX* transgenes (Figure S1a). The number of T‐DNA integration events was determined by Southern blot and showed that the 21 *35S* lines carrying both transgenes were genetically independent and included seven single insertion lines (Figure S1b). Of these, 14 *35S* lines (seven single insertion lines and seven multiple insertion lines) were selected for further evaluation. The segregating T_1_ generation was germinated on media containing hygromycin and resistant seedlings were transferred to soil for growth under greenhouse conditions. A TP309 wild‐type control and a transgenic line transformed with the empty vector, pCAMBIA1300 (p1300), were simultaneously grown under these conditions. To select the best performing transgenic lines, vitamin B_6_ content was evaluated in leaves of 45‐day‐old plants (vegetative stage) using a yeast bioassay for total vitamin B_6_ content (Figure S2a). All selected *35S* lines accumulated more vitamin B_6_ than the controls, with higher levels detected in single insertion lines in general, compared to multiple insertion lines (Figure S2b). Six single insertion *35S* lines (*35S*‐12a, *35S*‐12b, *35S*‐30, *35S*‐31, *35S*‐32, *35S*‐35) were selected for further characterization in the T_2_ generation. The measurement of total vitamin B_6_ content by yeast bioassay confirmed enhanced vitamin B_6_ levels in leaves of these lines, when compared with wild‐type (TP309) and empty vector control plants, as seen in the previous generation (Figure 2b, c). We also measured the vitamin B_6_ content in the roots of these lines and observed significant enhancement in five out of six of the *35S* lines (Figure 2d).

We next measured the vitamin B_6_ content in seeds of the transgenic rice lines. In the first instance, the content of mature fresh whole seeds (unpolished), polished seeds (removal of outer hull and aleurone layers) and isolated embryo were compared. In all cases, the highest vitamin B_6_ content was observed in the isolated embryos per unit fresh weight, and considerably less in polished versus unpolished seeds (Figure 2e). A statistically relevant increase (albeit modest) in vitamin B_6_ content could be observed in the embryos of all lines examined (Figure 2e). Similar increases were also observed in fresh unpolished seeds of most of the lines, and substantial vitamin B_6_ enhancement (up to 6.1‐fold) was observed in most of the fresh polished seeds compared with wild‐type and the empty vector control lines (Figure 2e). The content of dry unpolished and polished seeds was also determined. In all cases, the highest vitamin B_6_ content was observed in the mature dry unpolished seeds per unit dry weight (Figure 2f). Notably, a statistically significant increase in vitamin B_6_ levels was also observed in dry mature unpolished seeds of several of the *35S* lines (Figure 2f). However, the vitamin B_6_ content was not enhanced in the mature dry polished seeds from the transgenic lines compared to wild‐type (Figure 2f).

### Enhanced vitamin B_6_ content has only limited impacts on rice growth and development

**Figure 3:**
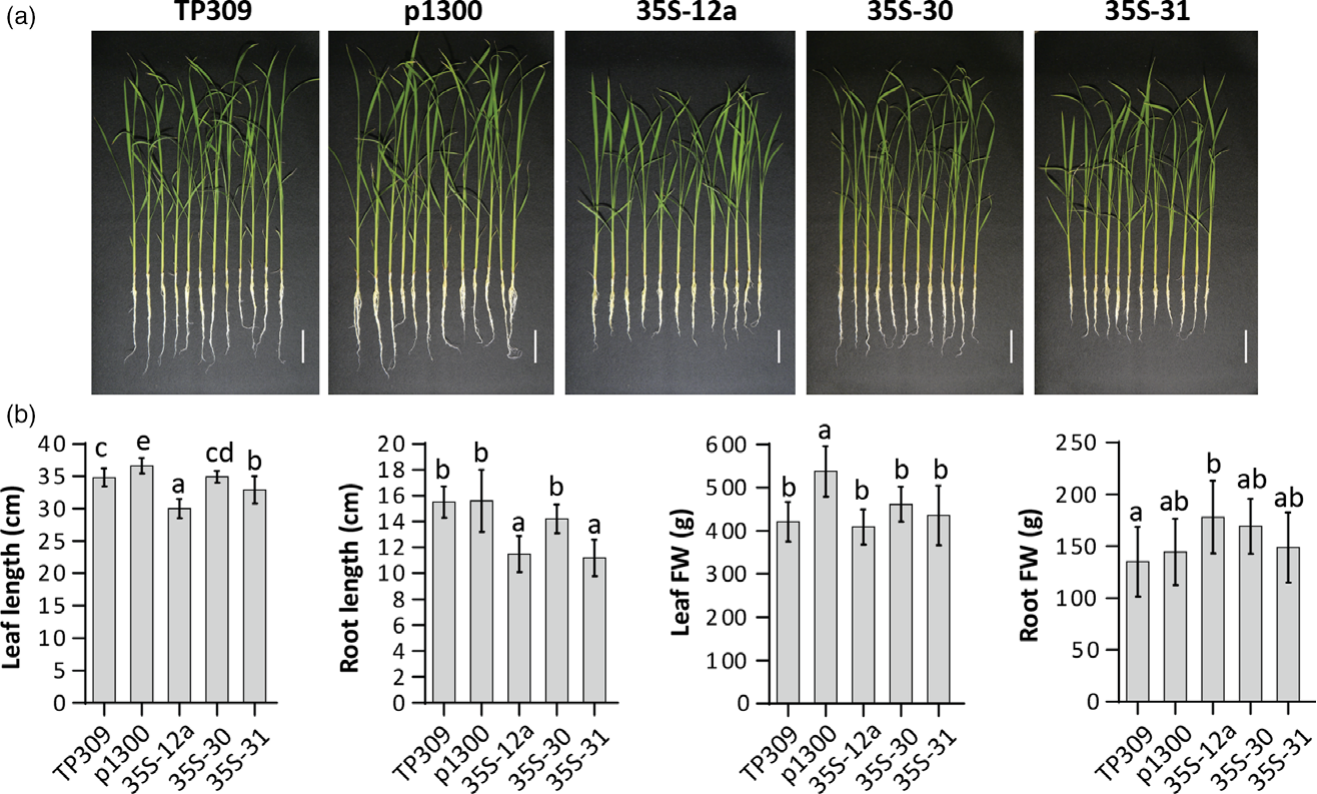
Phenotypic characterization of vitamin B_6_ enhanced rice lines. Plantlets in the T_3_ generation of the *35S* lines indicated were assessed for a phenotype compared to wild‐type (TP309) and the empty vector transgenic control (p1300) after growth for 15 days under hydroponic conditions. (a) Pictures are of plantlets at 15 days of growth under hydroponic conditions. The scale bar represents 5 cm. (b) Leaf length, root length, leaf fresh weight (FW) and root fresh weight of the rice lines. Average ± SD of 12 biological replicates. Tukey's multiple comparison test (*P *< 0.05).

The ability to increase vitamin B_6_ content in rice prompted us to examine its impact on growth and yield of rice. In the first instance, we characterized selected *35S* lines grown hydroponically in a climate chamber (28°C under a 16/8 h light/dark regime) for 15 days. Under these conditions, there were only limited (if any) impacts on the selected lines with respect to lengths and fresh weight of leaves and roots (Figure 3a,b). We also assessed mature plants of the *35S* lines grown under greenhouse conditions for effects on plant height, number of tillers and leaf dry weight, as well as the number of days from seed germination to panicle initiation (Table 1a). Evaluation of these parameters revealed a minor (8–13%) but statistically significant decrease in plant height of four of the *35S* lines (*35S*‐12a, *35S*‐31, *35S*‐32, and *35S*‐35) compared to the empty vector control plants. In contrast, the tiller number per plant, leaf dry weight, and time to panicle initiation were not altered (Table 1a). We also analyzed seed production parameters, specifically seed yield (based on weight), seed setting rate, number of panicles per plant and number of seeds per plant (Table 1b). There was no statistically significant difference in the number of panicles per plant and seed setting rate. The number of seeds per plant was decreased modestly in two lines (*35S*‐31 and *35S*‐32) compared to the empty vector control line. However, a statistically significant decrease in seed yield (17–42%) was observed in all transgenic lines compared to empty vector control plants, with the exception of line *35S*‐12b. The measured difference was mainly due to the reduced mass of 100 seeds for all yield‐affected lines.

**Table 1:** Phenotypic characterization of selected transgenic rice lines in the T_2_ generation. (a) Evaluation of selected 35S lines at maturity grown under greenhouse conditions (plant height, number of tillers per plant and leaf dry weight) and determination of the number of days from seed germination to the panicle initiation, compared to wild‐type (TP309) and the empty vector transgenic line (p1300). (b) Evaluation of seed production at maturity

a	Plant phenotype at maturity			Days to reach panicle initiation
	Plant height (cm)	Number of tillers/plant	Leaf dry weight (g)	
TP309	94.2 [±3.8]	4.7 [±0.9]	18.1 [±3.4]	115.3 [±4.8]
p1300	93.9 [±4.7]	5.0 [±1.0]	17.4 [±2.1]	111.1 [±6.0]
35S‐12a	87.2 [±2.1]**	5.3 [±1.0]	18.8 [±4.3]	108.6 [±4.6]
35S‐12b	91.1 [±5.6]	5.0 [±0.7]	15.9 [±3.1]	112.1 [±4.4]
35S‐30	92.4 [±4.1]	4.8 [±0.8]	18.0 [±3.9]	110.8 [±4.1]
35S‐31	81.6 [±2.4]**	5.0 [±1.1]	15.2 [±2.6]	108.8 [±3.4]
35S‐32	82.7 [±4.2]**	5.3 [±1.5]	14.3 [±5.7]	115.3 [±7.0]
35S‐35	86.3 [±6.2]*	5.8 [±0.7]	16.3 [±3.7]	111.9 [±4.1]

b	Plant seed production				
	Yield (g)	Seed setting rate (%)	Mass of 100 seeds (g)	Number of seeds/plant	Number of panicles/plant
TP309	7.4 [±1.8]	83.6 [±2.4]*	2.4 [±0.2]	332.8 [±64.2]*	4.6 [±0.7]
p1300	6.6 [±1.4]	75.4 [±9.0]	2.6 [±0.1]	261.0 [±53.0]	4.3 [±0.9]
35S‐12a	5.4 [±0.9]*	77.4 [±3.8]	2.1 [±0.0]**	256.4 [±39.1]	4.9 [±0.6]
35S‐12b	5.5 [±1.1]	66.7 [±8.6]	2.6 [±0.1]	216.8 [±49.1]	5.0 [±0.7]
35S‐30	5.0 [±1.1]*	78.1 [±6.5]	2.0 [±0.1]**	250.7 [±55.3]	4.4 [±0.9]
35S‐31	4.3 [±0.6]**	85.5 [±4.7]**	2.1 [±0.1]**	204.1 [±26.0]*	4.2 [±0.4]
35S‐32	3.8 [±1.6]**	82.2 [±7.2]	2.1 [±0.0]**	182.3 [±73.1]*	4.3 [±1.2]
35S‐35	5.5 [±1.0]*	79.9 [±7.7]	2.1 [±0.1]**	259.2 [±46.2]	5.0 [±1.0]

Average ± SD of nine biological replicates. Student's *t*‐test (p1300 versus transgenic line), *P < 0.05, **P < 0.01.

### Increased vitamin B_6_ content does not improve stress performance in rice

**Figure 4:**
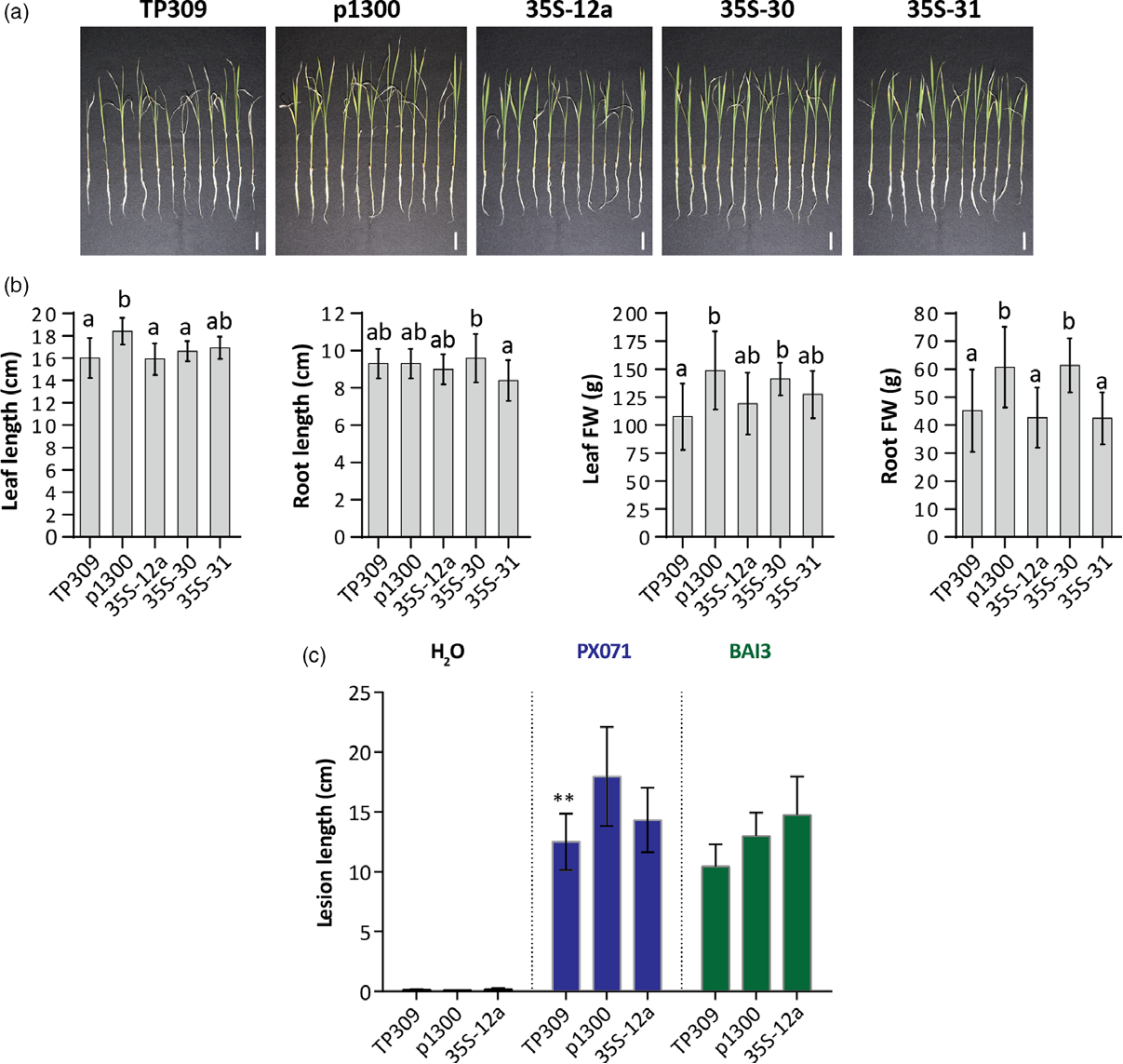
Phenotypic characterization of vitamin B_6_ enhanced rice lines under stress conditions. (a) Pictures of plantlets as indicated from the T_3_ generation grown in hydroponic culture 15 days after application of salt stress (150 mm NaCl). The scale bar represents 3 cm. (b) Leaf length, root length, leaf fresh weight (FW) and root fresh weight of *35S* lines in (a) compared to wild‐type (TP309) and the empty vector transgenic control (p1300). Average ± SD of 12 biological replicates. Tukey's multiple comparison test (*P* < 0.05). (c) Lesion length in rice leaves inoculated with *Xanthomonas oryzae*. Lesion length in leaves of 35S‐12a, T_3_ generation, 15 days after inoculation with PX071 (Leach *et al*., 1992) and BAI3 (Gonzalez *et al*., 2007a) *Xanthomonas oryzae* pv. *oryzae* strains compared to wild‐type (TP309) and the empty vector transgenic control (p1300). Inoculation with water was performed as a negative control. Average ± SD of four replicates for water and 8–12 replicates for bacterial inoculation (PX071‐TP309 (*n* =8), PX071‐p1300 (*n* = 7), PX071‐35S‐12a (*n* = 8), BAI3‐TP309 (*n* = 12), BAI3‐p1300 (*n* = 9), BAI3‐35S‐12a (*n* = 8)). Student's *t*‐test (p1300 versus transgenic line), **P < 0.01.

Given the considerable enhancement in vitamin B_6_ content of the generated transgenic rice plants, we next considered if stress tolerance was improved in these lines. To test for abiotic stress tolerance, we subjected 10‐day‐old hydroponically grown rice plantlets to salt stress for 15 days. While application of salt (150 mm sodium chloride) caused a decrease in plant growth in comparison with non‐stressed plantlets, there was no statistically significant phenotypic difference between controls (wild‐type TP309 and the empty vector control line p1300) and the selected transgenic lines accumulating higher levels of vitamin B_6_ (Figure 4a,b). To test for biotic stress tolerance, wild‐type TP309 and transgenic lines p1300 and *35S*‐12a (that accumulates high levels of vitamin B_6_ in leaves) grown under greenhouse conditions were inoculated with two *Xanthomonas oryzae* pv. *oryzae* strains, PX071 (Leach *et al*., 1992) and BAI3 (Gonzalez *et al*., 2007a), which both cause rice leaf blight. The evaluation of the lesion length 15 days post‐inoculation showed that all wild‐type and the transgenic line *35S*‐12a did not display a statistically significant difference in disease‐related symptoms compared to controls (Figure 4c).

### Vitamer profiling of rice lines with enhanced vitamin B_6_ content

**Figure 5:**
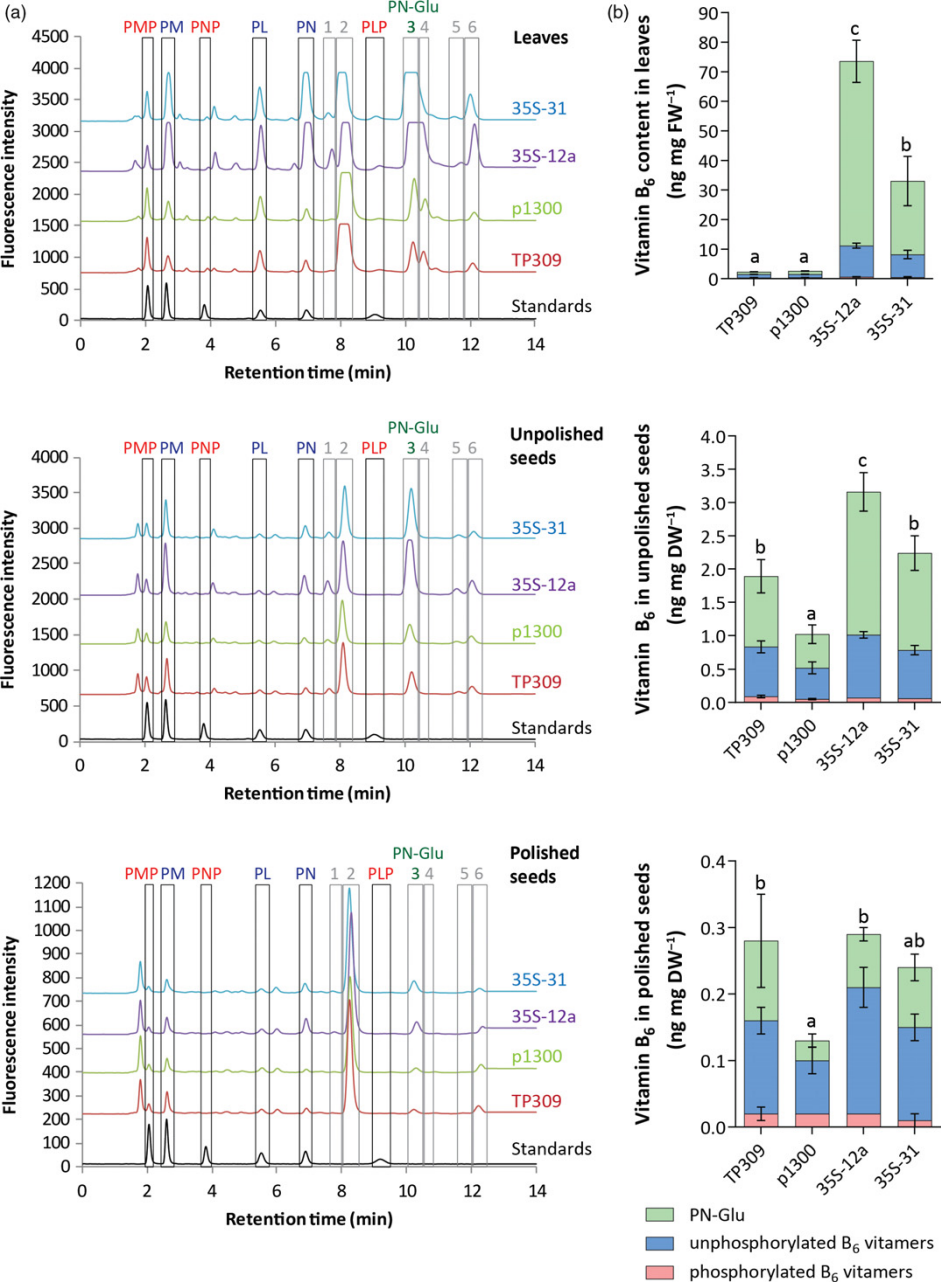
Profiling of B_6_ vitamers in rice lines enhanced in vitamin B_6_ content in the T_2_ generation. (a) HPLC chromatograms of leaf, unpolished seed and polished seed extracts of T_2_ generation plants grown in the greenhouse. Selected *35S* lines are compared with the wild‐type (TP309) and empty vector control (p1300). To facilitate the visualization, the profile of TP309 was offset by 700, p1300 by 1500, 35S‐12a by 2300 and 35S‐31 by 3100, relative to the baseline for the leaf extracts; the profile of TP309 was offset by 600, p1300 by 1300, 35S‐12a by 2000 and 35S‐31 by 2800, relative to the baseline for the unpolished seed extracts; the profile of TP309 was offset by 160, p1300 by 340, 35S‐12a by 500 and 35S‐31 by 670, relative to the baseline for the polished seed extracts. Profiles for the leaf samples 35S‐12a and 35S‐31 appear saturated for PN and PN‐Glu peaks but vitamin B_6_ quantification was performed on diluted samples having unsaturated signals. The numbers annotate peaks that do not correspond to the standards used. Peak 3 could be assigned as glucosylated pyridoxine (PN‐Glu) based on its correlation with a corresponding increase in PN content after treatment with β‐glucosidase (see Supplementary Figure S3). (b) Vitamin B_6_ content (PN‐Glu (green); unphosphorylated B_6_ vitamers (blue); phosphorylated B_6_ vitamers (pink)) in rice leaves, unpolished seeds and polished seeds according to the HPLC analysis. Average ± SD of four biological replicates. Tukey's multiple comparison test (*P *< 0.05) for total vitamin B_6_ content.

As outlined above, vitamin B_6_ is a family of compounds (some of which may have as yet undefined non‐coenzyme roles); we selected two *35S* lines (*35S*‐12a, *35S*‐31) for detailed B_6_ vitamer profiling, using a previously established HPLC method (Szydlowski *et al*., 2013; Li *et al*., 2015). The distribution of B_6_ vitamers was analyzed by HPLC in leaves, unpolished and polished seeds. In addition to peaks corresponding to the six standards used (PMP, PM, PNP, PN, PLP, PL), up to six peaks (numbered 1−6) with retention times different from the known standards could be observed in the plant extracts (Figures 5a and S3). As a large proportion of vitamin B_6_ can be stored in plants as β‐glucosides (Gregory, 1998), we treated rice leaf extracts with β‐glucosidase to determine if any of the unknown peaks could be assigned to glucosylated derivatives. All observed unknown peaks responded to the treatment (with the exception of peak 5, which was not detected in these leaf extracts), either decreasing (peaks 1/2 and 3) or increasing (peaks 4 and 6) in response to the treatment (Figure S3a). Peaks 1/2 and 3 were separately collected from untreated leaf extracts and treated with β‐glucosidase. Within the assigned 1/2 peak, peak 1 decreased in abundance upon treatment with β‐glucosidase (Figure S3b) but no new peak appeared and therefore could not be assigned to any known B_6_ vitamer with this method. Peak 2 did not respond to the β‐glucosidase treatment (Figure S3b). The disappearance of peak 3 with a concomitant increase in PN, allowed us to clearly assign this peak to glucosylated PN (PN‐Glu) (Figure S3b). We therefore assigned peak 3 as PN‐Glu equivalents and used it to calculate the abundance of this vitamer. A comparison of the chromatograms of the controls (wild‐type TP309 and the empty vector control line p1300) and transgenic rice plants (*35S*‐12a and *35S*‐31 lines) show clear differences in vitamer distribution in leaves and in unpolished seeds (Figure 5a). Vitamer quantification of these *35S* transgenic lines revealed that the main contributors to vitamin B_6_ accumulation in leaves and unpolished seeds were PN‐Glu (up to 57.8‐fold increase in leaves and 2.9‐ to 4.3‐fold increase in unpolished seeds) and the unphosphorylated forms (6.6‐ to 9.2‐fold increase in leaves and up to 2.0‐fold increase in unpolished seeds with a major contribution of PM and PN) (Figure 5b; Table 2). No increase was observed in phosphorylated forms except in leaves of the *35S*‐12a line, which showed a 1.7‐fold increase, primarily due to increased PLP levels (Table 2a). The transgenic lines did not display significantly higher accumulation of vitamin B_6_ in polished seeds as compared to the wild‐type control (Figure 5b; Table 2c), corroborating the results observed for the yeast bioassay (Figure 2). The major contributors to vitamin B_6_ content in polished seeds were PN‐Glu and unphosphorylated vitamers (Table 2c). With the HPLC technique, total vitamin B_6_ can be calculated as the sum of all quantified vitamers, including PN‐Glu as assigned in this study (Figure S3). Compared to the empty vector control line, the transgenic *35S* lines showed up to a 28.3‐fold increase in leaves, up to a 3.1‐fold increase in unpolished seeds and up to a 2.2‐fold increase in polished seeds (Figure 5b; Table 2). For polished seeds, it should be noted that the best performing line (*35S*‐12a) did not display a statistically significant increase compared to the wild‐type control TP309. Both TP309 and p1300 controls had similar vitamin B_6_ levels in leaves, but displayed a minor yet statistically significant difference in both unpolished and polished seeds (Figure 5b; Table 2). Moreover, the comparison of vitamin B_6_ levels in unpolished seeds (1.02 to 3.15 ng mg fresh weight (FW)^−1^) and polished seeds (0.13 to 0.29 ng mg FW^−1^) (Figure 5b; Table 2b,c) suggested that vitamin B_6_ mainly accumulates in the embryo and/or aleurone layer, corroborating the analysis of total vitamin B_6_ content using the yeast bioassay (Figure 2). These results are in agreement with previous studies reporting that other essential micronutrients are stored largely in the husk, aleurone and embryo of rice seeds (Kennedy *et al*., 2003; Lucca *et al*., 2006; Bhullar and Gruissem, 2013; Dong *et al*., 2016).

**Table 2:** HPLC analysis of B_6_ vitamers in leaves, unpolished seeds and polished seeds of wild‐type and transgenic rice. B_6_ vitamer distribution in leaves (a), dry unpolished seeds (b) and dry polished seeds (c) of the control and transgenic rice lines (T_2_ generation). Total unphosphorylated B_6_ vitamers corresponds to the PM, PN and PL contents for each replicate; total phosphorylated B_6_ vitamers correspond to the PMP, PNP and PLP contents for each replicate; total vitamin B_6_ corresponds to the unphosphorylated vitamers, the phosphorylated vitamers and the PN‐Glu contents for each replicate

Vitamers (ng mg FW^−1^)	Unphosphorylated					Phosphorylated					Glucosylated			
	PM	PN	PL	Total	Fold change	PMP	PNP	PLP	Total	Fold change	PN‐Glu	Fold change	Total vit. B_6_	Fold change
*a. Leaves*														
TP309	0.2^a^ [±0.02]	0.32^a^ [±0.06]	0.32^a^ [±0.02]	1.13^a^ [±0.04]	(×1.0)	0.13^a^ [±0.02]	nd	0.22^a^ [±0.05]	0.35^a^ [±0.06]	(×1.0)	0.85^a^ [±0.05]	(×0.8)	2.33^a^ [±0.05]	(×0.9)
p1300	0.20^a^ [±0.01]	0.31^a^ [±0.04]	0.64^a^ [±0.05]	1.15^a^ [±0.08]		0.13^a^ [±0.02]	nd	0.24^a^ [±0.05]	0.37^a^ [±0.05]		1.08^a^ [±0.06]		2.60^a^ [±0.17]	
35S‐12a	2.09^c^ [±0.16]	7.21^c^ [±0.81]	1.27^c^ [±0.01]	10.57^c^ [±0.85]	(×9.2)	0.11^a^ [±0.01]	nd	0.50^b^ [±0.09]	0.61^b^ [±0.08]	(×1.7)	62.37^c^ [±7.13]	(×57.8)	73.56^c^ [±7.47]	(×28.3)
35S‐31	1.49^b^ [±0.44]	5.13^b^ [±0.89]	1.05^b^ [±0.18]	7.67^b^ [±1.41]	(×6.6)	0.11^a^ [±0.02]	trace	0.35^a,b^ [±0.12]	0.51^a,b^ [±0.15]	(×1.4)	24.86^b^ [±8.36]	(×23.0)	33.04^b^ [±9.76]	(×12.7)
*b. Unpolished seeds*														
TP309	0.34^b^ [±0.03]	0.27^a,b^ [±0.08]	0.13^b^ [±0.01]	0.74^b,c^ [±0.09]	(×1.6)	0.07^c^ [±0.01]	trace	0.01^b^ [±0.00]	0.09^b^ [±0.02]	(×1.9)	1.06^a,b^ [±0.25]	(×2.1)	1.90^b^ [±0.35]	(×1.9)
p1300	0.21^a^ [±0.02]	0.16^a^ [±0.05]	0.09^a^ [±0.01]	0.47^a^ [±0.09]		0.04^a^ [±0.01]	nd	0.01^b^ [±0.00]	0.05^a^ [±0.01]		0.50^a^ [±0.14]		1.02^a^ [±0.20]	
35S‐12a	0.45^c^ [±0.02]	0.40^c^ [±0.03]	0.09^a^ [±0.00]	0.94^c^ [±0.05]	(×2.0)	0.06^b^ [±0.00]	trace	0.01^b^ [±0.00]	0.07^a,b^ [±0.00]	(×1.4)	2.15^d^ [±0.29]	(×4.3)	3.15^d^ [±0.30]	(×3.1)
35S‐31	0.33^b^ [±0.04]	0.29^b,c^ [±0.03]	0.09^a^ [±0.01]	0.72^b^ [±0.07]	(×1.5)	0.05^b^ [±0.00]	nd	0.01^a^ [±0.00]	0.06^a^ [±0.00]	(×1.2)	1.46^b,c^ [±0.26]	(×2.9)	2.23^b,c^ [±0.32]	(×2.2)
*c. Polished seeds*														
TP309	0.06^c^ [±0.01]	0.05^b^ [±0.01]	0.04^a^ [±0.00]	0.14^b^ [±0.02]	(×1.7)	0.01^b^ [±0.00]	nd	0.01^a^ [±0.00]	0.02^a^ [±0.01]	(×1.0)	0.12^b^ [±0.07]	(×3.6)	0.28^b^ [±0.10]	(×2.1)
p1300	0.03^a^ [±0.01]	0.02^a^ [±0.00]	0.03^a^ [±0.01]	0.08^a^ [±0.02]		0.01^a^ [±0.00]	nd	0.01^a^ [±0.00]	0.02^a^ [±0.00]		0.03^a^ [±0.01]		0.13^a^ [±0.02]	
35S‐12a	0.05^b,c^ [±0.01]	0.11^d^ [±0.01]	0.04^a^ [±0.01]	0.19^c^ [±0.03]	(×2.3)	0.01^a,b^ [±0.00]	nd	0.01^a^ [±0.00]	0.02^a^ [±0.00]	(×1.1)	0.08^a,b^ [±0.01]	(×2.5)	0.29^b^ [±0.02]	(×2.2)
35S‐31	0.04^a,b^ [±0.01]	0.06^b,c^ [±0.01]	0.04^a^ [±0.01]	0.14^b^ [±0.02]	(×1.6)	0.01^a^ [±0.00]	nd	0.00^a^ [±0.01]	0.01^a^ [±0.01]	(×0.7)	0.09^a,b^ [±0.02]	(×3.6)	0.23^a,b^ [±0.03]	(×1.8)

Average ± SD of four biological replicates. SD indicated in brackets. Tukey's multiple comparison test (*P* < 0.05). nd, not detected. trace: B_6_ vitamer content of at least one replicate could not be detected. Vitamin B_6_ content in wild‐type TP309 and in transgenic rice lines was compared with the empty vector control p1300 (fold change in brackets).

### Low transgene expression in seeds accounts for the limited increase in vitamin B_6_ content

**Figure 6:**
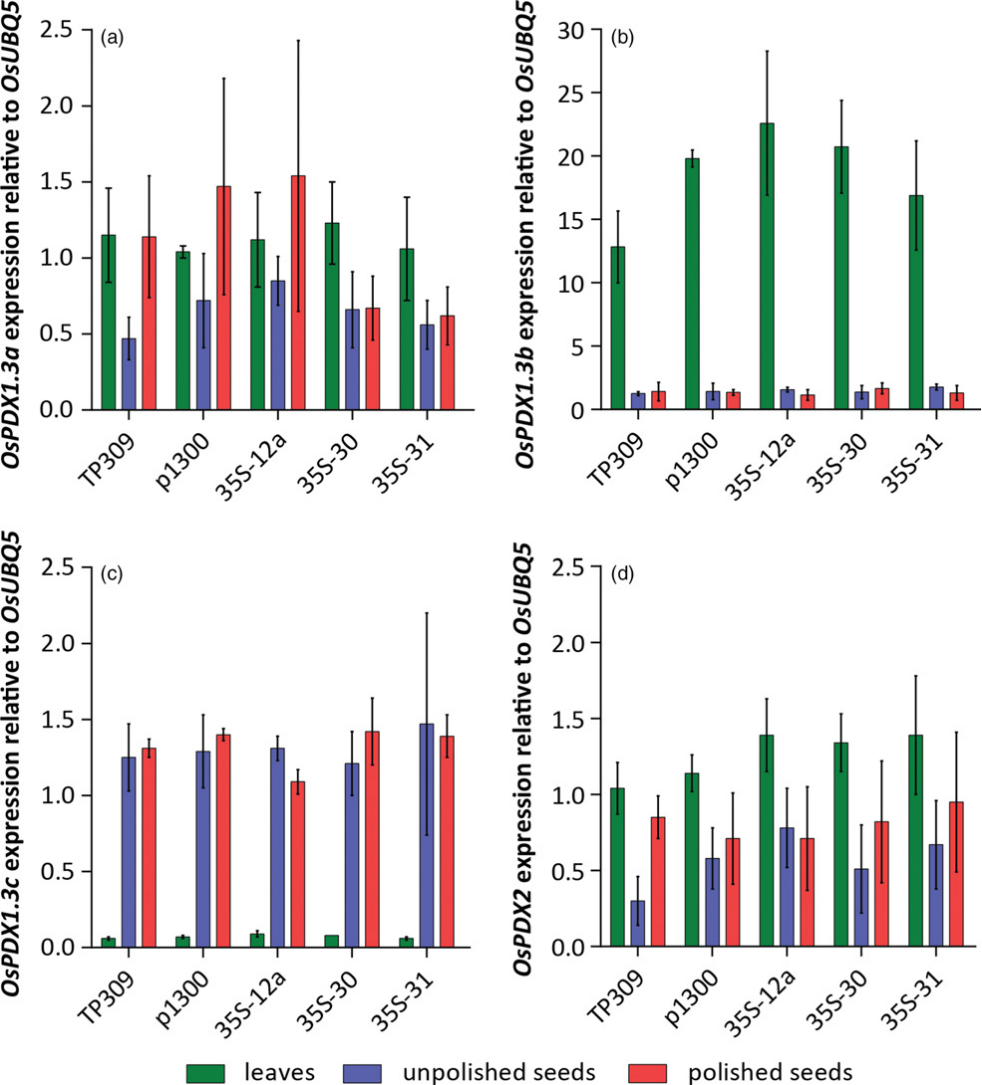
Real‐time quantitative PCR analysis of rice *PDX* gene expression in leaves, unpolished seeds, and polished seeds. Expression levels of Os*PDX1.3a* (a), Os*PDX1.3b* (b), Os*PDX1.3c* (c) and Os*PDX2* (d) in selected *35S* rice lines (T_2_ generation) compared with the wild‐type (TP309) and empty vector control line (p1300). Average ± SD of three biological replicates. Tukey's multiple comparison test (*P *< 0.05) for each sample type (i.e., leaves, unpolished seeds, polished seeds). Statistics are not shown as no significant difference was found.

Given the disparity between the ability to enhance vitamin B_6_ contents in rice leaves but not to the same extent in polished seeds in this study, we decided to further probe rice vitamin B_6_ biosynthesis. Recent studies have predicted three catalytic *PDX1* loci (Os7g01020, Os10g01080, and Os11g48080) in the rice genome (Moccand *et al*., 2014; Dell'Aglio *et al*., 2017). As they shared the highest similarity to Arabidopsis *PDX1*.*3*, they were annotated Os*PDX1.3a*‐*c*, respectively (Dell'Aglio *et al*., 2017). In the present study, we annotated the single homolog of *PDX2* in rice (Os2g03740), named hereafter Os*PDX2* (Figure 1). We analyzed the expression of Os*PDX1.3a*, Os*PDX1.3b*, Os*PDX1.3c* and Os*PDX2* in leaves, unpolished and polished seeds of wild‐type TP309, as well as selected *35S* transgenic lines and the empty vector control line. Interestingly, while Os*PDX1.3a* appears to be expressed ubiquitously in the tissues examined, Os*PDX1.3b* is substantially more abundant in leaves compared with seeds, whereas Os*PDX1.3c* is most abundant in seeds (Figure 6). These observations might suggest a specialization of Os*PDX1.3b* and Os*PDX1.3c* expression patterns in leaves and seeds, respectively. The expression of Os*PDX1.3b* was in agreement with publicly available expression data in Genevestigator (Hruz *et al*., 2008) (Figure S4) but the high expression of Os*PDX1.3c* in seeds was not noted in this resource. Os*PDX2* appears to be expressed at similar levels in all three tissue samples (Figure 6). In general, expression levels of the endogenous genes were unaltered in either leaves, or unpolished or polished seeds in the transgenic lines (Figure 6).

**Figure 7:**
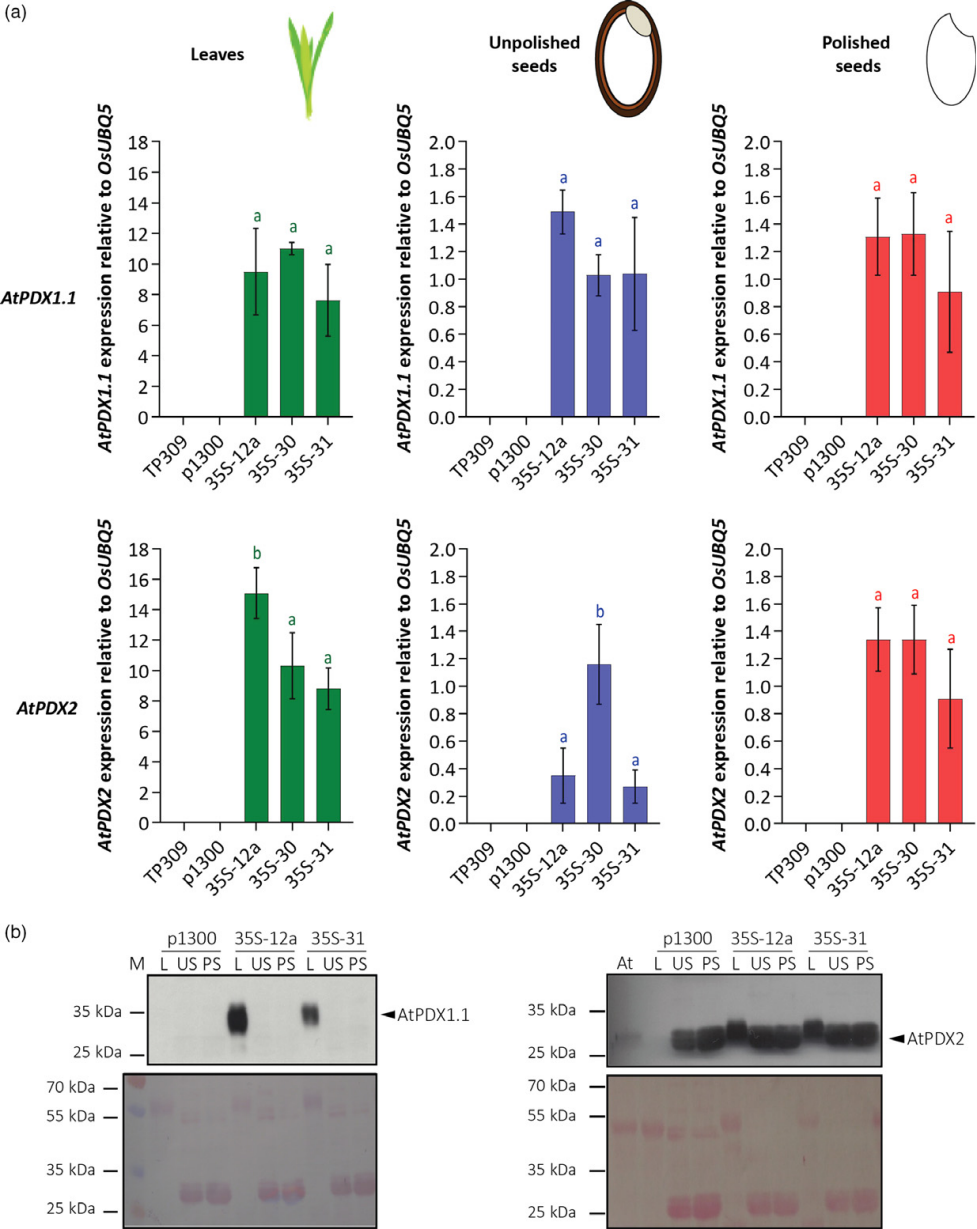
*PDX* transgene expression and protein accumulation in leaves, unpolished seeds, and polished seeds. (a) At*PDX1.1* and At*PDX2* transcript expression levels in *35S* transgenic rice lines (T_2_ generation) compared with wild‐type (TP309) and the empty vector control (p1300). Average ± SD of three biological replicates. Tukey's multiple comparison test (*P *< 0.05) for each tissue. (b) Western blot analysis of At*PDX1.1* and At*PDX2* protein abundance (upper panel) and Ponceau staining of the nitrocellulose membrane (lower panel) in control and transgenic rice lines. Here, 50 μg of total rice proteins and 30 μg of total Arabidopsis leaf proteins were probed with peptide antibodies specific to At*PDX1.1* (Raschke *et al*., 2011) or At*PDX2* (Tambasco‐Studart *et al*., 2007). M, protein molecular‐weight marker; L, leaves; US, unpolished seeds; PS, polished seeds; At, *Arabidopsis thaliana*.

As the endogenous rice *PDX* transcripts appeared to be unaltered, we were prompted to investigate the Arabidopsis transgene expression at both the transcript and protein level in an effort to explain the differential accumulation of vitamin B_6_. In the *35S* lines, while substantial transcript expression of Arabidopsis *PDX1.1* and *PDX2* was observed in all tissues examined, expression was higher (up to 10‐fold) in leaves compared with either unpolished or polished seeds (Figure 7a). We also examined protein levels using antibodies specific to Arabidopsis PDX1.1 and PDX2, respectively (Tambasco‐Studart *et al*., 2007; Raschke *et al*., 2011). Both Arabidopsis PDX1.1 and PDX2 could be clearly detected in leaves of the *35S* lines examined, immunostaining of which was not observed in the empty vector control line (Figure 7b). Interestingly, however, the Arabidopsis PDX1.1 protein could not be detected in unpolished or polished seeds of the transgenic lines (Figure 7b). While this may be accounted for by the lower transcript levels in these tissues, the inability to immunolabel the protein despite the high detection level in the leaves suggests that another mechanism contributes to the low level of protein in the seed tissue. The accumulation of PDX2 could not be deciphered due to cross‐reacting protein in these tissues (Figure 7b).

## Discussion

In the present study, we report vitamin B_6_ pathway engineering in a monocot species and its impact on plant growth and development. Considerable enhancement of vitamin B_6_ levels could be achieved by expressing the Arabidopsis *PDX1.1* and *PDX2* genes in rice. This enhancement was most pronounced in rice leaves but was more limited in seeds. This may be explained by the higher transcript levels of the At*PDX* genes observed in leaves rather than seeds. Indeed, the At*PDX1.1* and At*PDX2* proteins could be detected in leaves but not in seeds. Although, the *CaMV35S* promoter is considered to be less active in seed tissue compared with leaf tissue, there was a significant increase of At*PDX* transcripts in both unpolished and polished seeds from the selected transgenic rice lines and to similar levels. Yet, an increase in vitamin B_6_ was only observed in transgenic unpolished seeds and no significant enrichment could be detected in the rice endosperm. Previous studies show that transgenes driven by the *35S* promoter translate into detectable levels of the corresponding proteins in rice seeds (Furtado *et al*., 2008; Long *et al*., 2013). However, our immunochemical analyses failed to detect the Arabidopsis PDX proteins in either polished or unpolished seeds. This would seem to suggest that the accumulation of the protein is the limiting parameter for vitamin B_6_ production in this organ and may be most pronounced in the endosperm tissue. This observation warrants further investigation and could be suggestive of the fact that these proteins are tightly controlled (or repressed) in this tissue compared to leaves. In line with this hypothesis, we generated a small pool of rice lines expressing At*PDX1.1* and At*PDX2* under the control of the endosperm‐specific *globulin* (*Glob*) promoter (included in Figure S1). We could observe transgene expression in these *Glob* lines in seed tissue (Figure S5a, b), and notably at similar levels to those observed in the *35S* lines (compare with Figure 7a). By contrast, expression of the At*PDX* transgenes was not detected in leaf tissue of the rice *Glob* lines, as expected (Figure S5a, b). However, as for the *35S* lines, the AtPDX proteins could not be detected in seed tissue of the *Glob* lines (Figure S5c, d). Moreover, while we did observe an increase in vitamin B_6_ content in the seeds of the *Glob* lines generated (albeit a small pool) (Figure S5e, f), the increase was similar to that observed in the seeds of the *35S* lines (Figure 2e, f). Also similar to the *35S* lines, the levels of vitamin B_6_ in polished seeds of the *Glob* lines was not above the wild‐type level (Figure S5e, f). As expected there was no increase in vitamin B_6_ content in the leaves or roots of the *Glob* lines compared with the control lines (Figure S5g, h). Although a more rigorous study with the use of endosperm‐specific promoters is required in the future, our analysis here suggests that enhancing seed vitamin B_6_ content could be constrained by the ability to express the PDX1 proteins and may indicate an endogenous regulatory mechanism of these proteins in rice seeds. Of course, a potentially limited availability of precursors in endosperm tissue might also represent another bottleneck for upregulated vitamin B_6_ biosynthesis therein.

Increasing vitamin B_6_ levels had very little impact on rice growth and development. Nonetheless, a significant yield penalty was observed by assessing the mass of 100 seeds in selected transgenic rice lines. Expression of *PDX* transgenes in other plant species, including Arabidopsis, cassava and potato (Raschke *et al*., 2011; Li *et al*., 2015; Bagri *et al*., 2018) was not reported to be associated with yield penalty. However, previous work in Arabidopsis showed a positive correlation between vitamin B_6_ content and seed size, yet a yield penalty in terms of number of seeds was observed (Raschke *et al*., 2011). Recent work in maize indicated that vitamin B_6_ is essential for embryo development but has a limited role in endosperm development (Yang *et al*., 2017), therefore the mechanism behind the observations in rice may not be restricted to monocots and may be species‐dependent. The vitamin B_6_ biosynthesis *de novo* pathway in rice has so far only been partially described. Based on homology with genes previously discovered in Arabidopsis (Tambasco‐Studart *et al*., 2007; Raschke *et al*., 2011), there are three homologs for *PDX1* in rice (*PDX1.3a‐c*) (Dell'Aglio *et al*., 2017), and we identified a single homolog for *PDX2*. The expression profiles of Os*PDX1.3b* and Os*PDX1.3c* in leaves and in seeds, respectively, suggest organ‐specific expression for these two homologs. The expression level of Os*PDX1.3b* in photosynthetic tissue (i.e., leaf) was considerably higher than the other two *PDX1.3* genes. It is of note in the present study, that while we demonstrate that At*PDX1.1* and At*PDX2* from Arabidopsis are functional in rice based on the significant increase in total vitamin B_6_ content in leaves, they have little impact on the endogenous *PDX* gene expression levels in rice.

The salt stress assays carried out here suggest that increased vitamin B_6_ content in unpolished seeds and leaves of transgenic rice lines does not impact their performance under these conditions. Similarly, disease symptom evaluation upon inoculation with the bacterial leaf blight pathogen *Xanthomonas oryzae* pv.* oryzae* did not reveal significant differences between control lines and a transgenic line accumulating high levels of vitamin B_6_. This contrasts with the previously reported positive contribution of vitamin B_6_ to plant responses to environmental stress in various other species, which were notably members of the eudicot group (Arabidopsis, tomato) (Raschke *et al*., 2011; Zhang *et al*., 2014, 2015). However, it should be noted that the evidence for vitamin B_6_ contribution to plant performance under biotic and abiotic stresses mostly comes from mutant lines or virus induced gene‐silenced lines impaired in vitamin B_6_ biosynthesis (Titiz *et al*., 2006; Zhang *et al*., 2014, 2015). It remains to be determined whether the positive contribution of vitamin B_6_ to stress responses *in planta* is due to a direct effect based on its reported antioxidant properties and/or an indirect effect based on its role as a coenzyme, possibly for an enzyme contributing to antioxidant capacities (Fitzpatrick, 2011). Whether the non‐responsiveness to environmental stress observed here is species or group (eudicots versus monocots)–dependent would require analysis in other monocot and eudicot plant species with enhanced vitamin B_6_ content.

Interestingly, the B_6_ vitamer profiles of rice wild‐type TP309 leaves determined in this study differ from those measured in cassava wild‐type cv.60444 leaves (Li *et al*., 2015). In particular, the contribution of PN‐Glu to total vitamin B_6_ is lower for rice leaves (36%) as compared to cassava leaves (57%). Noticeably, PN‐Glu represents a major proportion of vitamin B_6_ pools in rice unpolished (56%) and polished (43%) seeds. The profiles of B_6_ vitamers in transgenic cassava (Li *et al*., 2015) and rice (this study) upon ectopic expression of At*PDX1.1* and At*PDX2* transgenes indicate that the increase in vitamin B_6_ biosynthesis translates into similar accumulation of B_6_ vitamers, that is, PN‐Glu also represents a major accumulating form of vitamin B_6_ (Table 2). The function of PN‐Glu *in planta* is unknown, but it is suggested to be an unreactive/inert form of vitamin B_6_ (Gregory, 1998) and its preferential accumulation in these transgenic rice and cassava lines suggests that it could participate in the preservation of vitamin B_6_ homeostasis. Indeed, balancing of B_6_ vitamers has recently been demonstrated to be essential for normal plant development (Colinas *et al*., 2016). Due to the reduced bioavailability of glucosylated vitamers in humans (approximately 50%, Gregory, 2012), the identification of genetic factors and conditions controlling the interconversion of glucosylated vitamers to the non‐glucosylated equivalents will be of high importance for future strategies targeted toward B_6_ vitamin biofortification of crops. In general, there have been contrasting results for metabolic engineering of B vitamins in rice endosperm. Transgenic rice expressing folate (vitamin B_9_) biosynthetic genes from Arabidopsis (*AtGTPCHI* and *AtADCS*) under the control of *Glob* and *glutelin B*_*1*_ promoters, respectively, displayed a substantial increase of folate content in rice endosperm (Storozhenko *et al*., 2007), which was further accentuated in a later study by the expression of a synthetic folate binding protein to enhance stability (Blancquaert *et al*., 2015). In contrast, constitutive overexpression of endogenous vitamin B_1_ biosynthesis genes (*OsTHIC* and *OsTHI1*) in rice led to significant increases in leaves and to a more limited extent in unpolished seed, but most of the gain was lost during polishing (Dong *et al*., 2016). While identifying bottlenecks in vitamin B_6_ biosynthesis in the rice endosperm should be prioritized, future strategies to enrich vitamin B_6_ content in rice endosperm might also benefit from the identification and characterization of vitamin B_6_ transporters, as well as other factors contributing to vitamin accumulation and stabilization, such as vitamin B_6_ binding proteins.

In the present study we demonstrated the potential of the *PDX* transgene approach to considerably enhance the vitamin B_6_ content in rice and with no impact on endogenous *PDX* gene expression or overall growth and development, with the exception of a modest impact on seed yield. This study in a monocot species has provided some interesting insights into potential regulatory pathways of vitamin B_6_ sequestration in plants and opened up areas for future fundamental investigation as well as applied studies that will enable significant vitamin B_6_ enrichment in rice endosperm.

## Experimental procedures

### Binary vector construction

The constitutive expression vector containing *p35S*::At*PDX1.1*‐*p35S*::At*PDX2* (*Arabidopsis thaliana PDX1.1*, At2g38230 and *PDX2*, At5g60540) previously described (Li *et al*., 2015) was used in this study. The endosperm‐specific expression vector containing *pGlob*::At*PDX1.1*‐*pGlob*::At*PDX2* was generated by substituting the cauliflower mosaic virus (*CaMV*) *35S* promoters in p35S::At*PDX1.1*‐p35S::At*PDX2* with a 1.0 kb *Globulin* (*Glob*) promoter sequence from rice (le Qu and Takaiwa, 2004). Both expression vectors were constructed in the pCAMBIA1300 backbone. A schematic representation of the T‐DNA region is provided in Figure 2.

### Rice transformation and molecular characterization of transgenic lines

Binary vectors were introduced into *Agrobacterium tumefaciens* strain EHA105 (Hood *et al*., 1993) and rice transformation, selection on hygromycin and regeneration were conducted according to a previously established protocol (Nishimura *et al*., 2006). Isolation of genomic DNA from rice leaves was performed based on a previously established protocol (Sheu *et al*., 1996). Frozen ground tissue was suspended in 900 μL of urea extraction buffer (without sarkosine) and extracted with 1 volume of phenol:chloroform:isoamylalcohol (25:24:1, pH 7.5–8.0) at room temperature for 15 min. The recovered aqueous phase was mixed with 0.1 volume of 3 m sodium acetate (pH 5.2), 1 μL RNase A (20 mg mL^−1^) and 1 volume of isopropanol. The extract was incubated for 20 min at −80°C and centrifuged at 16 100 ***g*** for 3 min at room temperature. The DNA pellet was washed with 70% followed by 100% ethanol, vacuum dried and re‐suspended in sterile Milli‐Q water. The identification of transformants containing both At*PDX1.1* and At*PDX2* transgenes was done by PCR. Primers are detailed in Table S1. Southern blot analyses were conducted for the confirmation of T‐DNA insertion and determination of transgene copy number, using a DIG‐dUTP‐labeled *hptII* probe, synthesized by PCR (PCR DIG probe synthesis kit; Roche Diagnostics AG, Risch‐Rotkreuz, Switzerland) (Table S1), following a previously described procedure (Vanderschuren *et al*., 2009).

### Plant material

Dried seeds of *Oryza sativa* ssp. *japonica* cv. Taipei 309 were de‐husked and surface‐sterilized in 70% ethanol for 30 sec, then in a 1.5% sodium hypochlorite solution including 0.01% Tween 20 for 30 min under vigorous shaking. Surface‐sterilized seeds were rinsed five times with sterile water before being placed in sterile plastic jars containing Murashige and Skoog (MS) medium (1× MS salts including vitamins (Duchefa, Haarlem, Netherlands), 3% (w/v) sucrose, 0.3% (w/v) gelrite; pH 5.8) containing 50 mg L^−1^ of hygromycin (Carl Roth, Karlsruhe, Germany) for selection of transgenic lines. Seeds were stratified in the dark at 28°C for 48 h before being transferred to a climate chamber (28°C under a 16/8 h light/dark regime) for 12 days. Plantlets were subsequently transferred to soil and grown under greenhouse conditions (12 h light at 30°C and 70% humidity, 12 h dark at 20°C and 60% humidity). Either T_2_ or T_3_ generation plants were used for analyses, as indicated. Nine replicates per line were distributed in three pots of three replicates, organized in three blocks in the greenhouse. The three or four replicates analyzed came from the three blocks and the different tissues were sampled as follows: leaves, three leaves from three different tillers of 45‐day‐old plants (vegetative stage) were pooled and frozen in liquid nitrogen; roots, during harvest, root samples were collected, rinsed with tap water and frozen in liquid nitrogen; fresh mature seeds, 1–2 weeks before harvesting, mature seeds were sampled. Some seeds were de‐husked only (referred to as fresh unpolished seeds) and frozen in liquid nitrogen. Other seeds were de‐husked and the aleurone layer was peeled off using forceps and a razor blade (Kuwano *et al*., 2011). The inner starchy endosperm (referred to as fresh polished seeds) and the embryo were collected separately and frozen in liquid nitrogen. Dry mature seed samples were collected from harvesting fully ripened panicles and drying for 5 days at 37°C. The dried seeds were either de‐husked only (referred to as dry unpolished seeds) and stored at −80°C or de‐husked and polished for 2 min (seed polisher PEARLEST, Kett) to obtain the starchy endosperm (referred to as dry polished seeds) and stored at −80°C.

### Stress treatments

#### Salt stress

For the salt stress treatment, de‐husked seeds were germinated in water for 4 days, including 48 h in the dark to synchronize germination. Rice seedlings were then hydroponically grown in Yoshida's solution (0.7 mm K_2_SO_4_, 2 mm Ca(NO_3_)_2_, 0.1 mm KH_2_PO_4_, 0.5 mm MgSO_4_, 0.1 mm KCl, 10 μm H_3_BO_3,_ 0.5 μm MnSO_4_, 0.2 μm CuSO_4_, 0.01 μm (NH_4_)Mo_7_O_24_, 0.5 μm ZnSO_4_, 100 μm Fe‐EDTA) (Kobayashi *et al*., 2005) in a climate chamber (28°C under a 16/8 h light/dark regime) for 6 days. Four concentrations of NaCl (50, 100, 150, 200 mm) were initially evaluated. The concentration of 150 mm NaCl was used in this study because of the intermediate visible effects on rice plantlet morphology. Salt stress treatment was applied for 15 days using Yoshida's solution supplemented with 150 mm NaCl, with changes of the solution every 2 days. Plantlets were grown simultaneously in Yoshida's solution without salt as controls.

#### Leaf blight assay

*Xanthomonas oryzae* pv.* oryzae* pathogenicity assays were performed under greenhouse conditions (12 h light at 28°C and 80% humidity, 12 h dark at 25°C and 70% humidity). TP309 wild‐type and transgenic line 35S‐12a were grown for 4–5 weeks and inoculated with two different *Xanthomonas oryzae* pv.* oryzae* strains: PX071 (Leach *et al*., 1992) and BAI3 (Gonzalez *et al*., 2007a). Leaf clip inoculation was performed as previously described (Kauffman *et al*., 1973) with bacterial suspensions grown up to a cell density value of OD_600 nm_ = 0.2. Lesion length was measured 15 days post‐inoculation.

### Vitamin B_6_ quantification

#### Yeast bioassay

Yeast bioassays for vitamin B_6_ content were performed according to a method previously established with the *Saccharomyces carlsbergensis* American Type Culture Collection 9080 strain (Tambasco‐Studart *et al*., 2005). The amount of plant material required for vitamin B_6_ extraction varied between organs: leaves (50 mg), roots (100 mg), dry seeds (50 mg), fresh seeds (20 mg), embryo (20 mg). Frozen ground tissues were re‐suspended in 20 mm sulfuric acid (ratio: 100 mg tissue/1 mL extraction buffer), incubated for 30 min in the dark at room temperature and the extract was sterilized for 1 h at 100°C. After extraction, the solution was adjusted to pH 5.7 using 3 m sodium acetate and centrifuged. The supernatant was then treated with acid phosphatase (0.2 U/10 μL in 50 μL plant extract) (Sigma‐Aldrich, St. Louis, MI, USA) and β‐glucosidase (0.2 U/10 μL in 50 μL plant extract) (Sigma‐Aldrich, St. Louis, MI, USA) for 12–15 h at 37°C to convert phosphorylated and glucosylated forms of vitamin B_6_ to free forms. Total vitamin B_6_ content was calculated using the linear range of a dose−response curve constructed with known amounts of commercial pyridoxine hydrochloride (Sigma‐Aldrich, St. Louis, MI, USA).

#### High‐performance liquid chromatography measurement

Quantification of B_6_ vitamers was performed essentially according to a previously established HPLC method with minor modifications (Szydlowski *et al*., 2013). B_6_ vitamers were extracted from frozen ground tissues using 50 mm ammonium acetate pH 4.0 (ratio: 100 mg tissue/200 μL ammonium acetate). The extract was vortexed for 10 min, centrifuged at 16 100 ***g*** for 15 min at room temperature and the supernatant incubated for 3 min at 99°C. The extract was again centrifuged at 16 100 ***g*** for 15 min at room temperature and the supernatant was used for analysis. The extract was analyzed on an Agilent Technologies 1200 HPLC instrument to determine B_6_ vitamer profiles, using a Sunfire C18 column (Waters, Milford, MA, USA), 4.6 × 150 mm, 3.5 μm particle diameter. The chromatography and detection method used was previously described (Szydlowski *et al*., 2013). The amount of glucosylated PN vitamer (PN‐Glu) was extrapolated from the increase in PN content observed after treatment of an equal amount of the original extract with β‐glucosidase (Sigma‐Aldrich, catalog number 49 290, 10 μL of 15 mg mL^−1^ stock dissolved in 50 mm ammonium acetate pH 4.0) for 2–4 h at 37°C and then boiled as above, prior to injection. Vitamer contents were calculated using the linear range of standard curves constructed with known amounts of PN, PM, PL, PNP, PMP and PLP (Colinas *et al*., 2016). Measurements for vitamers showing saturated signals on the largest injection volume were re‐injected with a smaller volume and/or diluted prior to analysis.

### Gene expression quantification using real‐time quantitative PCR (RT‐qPCR)

#### RNA extraction

Total RNA from leaves was extracted according to a previously established method with some modifications (Chang *et al*., 1993). Frozen ground tissue was mixed with 1 mL of extraction buffer (2% cetyl trimethylammonium bromide, 2% polyvinylpyrrolidone K‐30, 100 mm Tris HCl, 25 mm ethylenediaminetetraacetic acid, 2 m NaCl, 0.5 g L^−1^ spermidine) and 2% β‐mercaptoethanol, incubated at 50°C with shaking at 400–500 rpm for 15 min and extracted twice with 1 volume of chloroform:isolamylalcohol (24:1, pH 7.5–8.0). Nucleic acids from the recovered aqueous phase were precipitated with 1 mL of ice cold absolute ethanol for 30 min at −80°C and centrifuged at 16 100 ***g*** for 30 min at 4°C. The pellet was washed with 80% ethanol and re‐suspended in diethylpyrocarbonate (DEPC)‐treated water. RNA was precipitated in 2 m lithium chloride overnight at −20°C and centrifuged at 16 100 ***g*** for 30 min at 4°C. The RNA pellet was washed with 80 and 100% ethanol, vacuum dried and re‐suspended in DEPC‐treated water.

RNA extraction from fresh rice seeds was performed using an established protocol (Singh *et al*., 2003).

#### RT‐qPCR analysis

cDNA was synthesized using random hexamer oligonucleotide primers with the RevertAid First Strand cDNA Synthesis Kit (Thermo Fisher Scientific AG, Basel, Switzerland) according to the manufacturer's instructions, with 2 μg total RNA for leaves and 3 to 5 μg total RNA for fresh seeds. RT‐qPCR reactions were performed using the LightCycler 480 II (Roche Diagnostics AG, Risch‐Rotkreuz, Switzerland) system and Fast SYBR^®^ Green Master Mix (Applied Biosystems, altham, MA, USA, Thermo Fisher Scientific, Basel, Switzerland). The reaction mixture contained 1 μL of DEPC‐treated water, 1 μm of each primer, 5 μL SYBR master mix and 2 μL cDNA diluted 10‐fold for leaves and undiluted for seeds. PCR conditions were as follows: initial denaturation for 2 min at 95°C followed by 40 cycles of denaturation for 10 sec at 95°C, annealing for 20 sec at 60°C and extension for 30 sec at 72°C. Relative target gene expression levels were normalized to the rice reference gene *UBQ5* (Jain *et al*., 2006) and calculated using the delta delta CT method (Livak and Schmittgen, 2001). Sequences of primers used for RT‐qPCR analysis are detailed in Table S2.

#### In silico identification of the rice PDX2 gene

The rice ortholog of *Arabidopsis thaliana *At*PDX2* (At5g60540) was identified by BLASTing the Arabidopsis protein sequences from the TAIR10 database (Lamesch *et al*., 2012) against the available translated *Oryza sativa* v7_JGI genome in Phytozome (Ouyang *et al*., 2007).

### Immunochemical analyses

Protein extraction was performed according to a previously established protocol with some modifications (Svozil *et al*., 2014). Total proteins were extracted from frozen ground tissues in 1 mL sodium dodecyl sulfate (SDS) buffer (4% SDS, 40 mm Tris‐base, 5 mm MgCl_2_, and 2 × protease inhibitor mix (Roche, Basel, Switzerland)) for 20 min at room temperature. Cell debris was centrifuged for 15 min at 16 100 ***g*** and the supernatant transferred to a fresh tube. Protein concentration was determined using the Pierce^™^ BCA Protein Assay Kit (Thermo Fischer Scientific). Immunodetection was based on a previously established protocol for Arabidopsis (Titiz *et al*., 2006), including some modifications. Proteins were separated on 12% SDS‐PAGE gels and subsequently transferred onto nitrocellulose membranes. Membranes were blocked in Tris‐buffer saline solution containing 0.05% Tween‐20 and 20% milk powder overnight at 4°C. At*PDX1.1* protein was detected with specific antibodies raised against the peptide GEGAMTETKQKSP (Raschke *et al*., 2011) and At*PDX2* protein was detected with antibodies raised against the recombinant Arabidopsis protein (Tambasco‐Studart *et al*., 2007). Blots were incubated with primary antibodies (1:1000 dilutions) for 1 h and then probed with a Goat Anti‐Rabbit IgG (H+L)‐HRP conjugated secondary antibody (Bio‐Rad, Hercules, CA, USA) (dilution 1:5000) for 1 h, before chemiluminescent detection.

### Statistical analyses

Detailed description of statistical analysis is provided in Data S1.

## Data statement

Supporting data can be accessed as Supplementary Information on The Plant Journal website.

## Conflict of interest

The authors declare no conflict of interest.

## Supporting information


**Figure S1.** Molecular characterization of the generated dual‐expressing At*PDX1.1* and At*PDX2* transgenic rice lines in the T_0_ generation.
**Figure S2.** Analysis of total vitamin B_6_ contents of transgenic rice lines in the T_1_ generation.
**Figure S3.** Assignment of a glucosylated B_6_ vitamer in rice leaf extracts.
**Figure S4.** Rice *PDX* gene expression patterns across different tissues.
**Figure S5.** *PDX* transgene expression, protein accumulation and vitamin B_6_ content in the rice *Glob* lines.

**Table S1.** Primers used for the molecular characterization of generated transgenic rice lines.
**Table S2.** Primers used for real‐time quantitative PCR analysi*s*.

**Data S1**. Statistical analysis.
