# Supplementary material for: Enhancement of vitamin B6 levels in rice expressing Arabidopsis vitamin B6 biosynthesis de novo genes
Source: Plant J. 2019 Jul 11;99(6):1047–65. doi: 10.1111/tpj.14379 (PMC6852651; doi:10.1111/tpj.14379)
Supplement: Supplementary file 2 — Table S1. Primers used for the molecular characterization of generated transgenic rice lines. Table S2. Primers used for real‐time quantitative PCR analysis. [file TPJ-99-1047-s002.pdf]

Table S1. Primers used for the molecular characterization of generated transgenic rice lines.

Target gene	Gene reference	Primer name	Primer sequence (5'-3')
***AtPDX1.1* (construct)**	At2g38230	AtPDX1.1-CDS-F<br>AtPDX1-plasmid-R	GTGAGGAGTGTGAACGGAGC<br>ACAATCAGTAAATTGAACGGAG
***AtPDX2* (construct)**	At5g60540	AtPDX2-CDS-F<br>AtPDX2-plasmid-R	GGCAGACAGAGCAGTTGGTC<br>GGAACTACTCACACATTATTCTGGAG
hptII	-	hptII-F<br>hptII-R	TCTCGATGAGCTGATGCTTTGG<br>AGTACTTCTACACAGCCATCGG

Table S2. Primers used for real-time quantitative PCR analysis.

Target gene	Gene reference	Primer name	Primer sequence (5'-3')
AtPDX1.1	At2g38230	AtPDX1.1t-F<br>AtPDX1.1t-R	GATGATAAGGTTGAGAGGTTCGC<br>GCATATCTCATTAAAGCAGGGC
AtPDX2	At5g60540	AtPDX2t-F<br>AtPDX2t-R	GCTCTACGTGAGTTTGTTAAG ATG<br>CTCTTTCTGACCAACTGCTC
OsPDX1.3a	LOC_Os07g01020	OsPDX1.3a-F<br>OsPDX1.3a-R	GCCTTATCCTTCTTTCGCTA<br>CTGGCTGGCTGGTGTCTAATTC
OsPDX1.3b	LOC_Os10g01080	OsPDX1.3a&1.3b-F<br>OsPDX1.3b-R	TTGGCATCAACCTCTCCG<br>AGGAAGGATGGCACTCTACG
OsPDX1.3c	LOC_Os11g48080	OsPDX1.3c-F<br>OsPDX1.3c-R	GTCGGCATCAACCTCAACG<br>AGAGGGAGCAGGAGCAGGA
OsPDX2	LOC_Os02g03740	OsPDX2-F<br>OsPDX2-R	CCCATCTTCGAGTAGCTCATG<br>CTCAACTTTCTCTATTGTCACC
OsUBQ5	LOC_Os01g22490	UBQ5-F<br>UBQ5-R	ACCACTTCGACCGCCACTACT<br>ACGCCTAAGCCTGCTGGTT
