# Supplementary material for: Enhancement of vitamin B6 levels in rice expressing Arabidopsis vitamin B6 biosynthesis de novo genes
Source: Plant J. 2019 Jul 11;99(6):1047–65. doi: 10.1111/tpj.14379 (PMC6852651; doi:10.1111/tpj.14379)
Supplement: Supplementary file 3 — Data S1. Statistical analysis. [file TPJ-99-1047-s003.docx]

**Data presentation in Figure 5**

To facilitate the visualization, the profile of TP309 was offset by 700, p1300 by 1500, 35S-12a by 2300, 35S-31 by 3100, relative to the baseline for the leaf extracts; the profile of TP309 was offset by 600, p1300 by 1300, 35S-12a by 2000 and 35S-31 by 2800, relative to the baseline for the unpolished seed extracts; the profile of TP309 was offset by 160, p1300 by 340, 35S-12a by 500 and 35S-31 by 670, relative to the baseline for the polished seed extracts. Profiles for the leaf samples 35S-12a and 35S-31 appear saturated for PN and PN-Gly peaks but vitamin B_6_ quantification was performed on diluted samples having unsaturated signals. The numbers annotate peaks that do not correspond to the standards used. Peak 3 could be assigned glycosylated pyridoxine (PN-Gly) based on its correlation with a corresponding increase in PN content after treatment with Beta-glycosidase (see Supplementary Figure 3).

**Statistical analysis**

The effects of heterologous expression of *AtPDX1.1* and *AtPDX2* on B_6_ vitamer content, on gene expression levels and on phenotype in response to salt stress were evaluated by one-way analysis of variance (ANOVA) at the 0.05 significance level. When the effect was significant, the ANOVA was followed by a Tukey’s test for *post-hoc* pairwise comparisons (α = 0.05). Normality was assessed using the Shapiro-Wilk’s test on residuals (α = 0.01), and homoscedasticity was tested using the Bartlett’s test (α = 0.01). Total vitamin B_6_, PM, PN, PL, unphosphorylated B_6_ vitamer and PN-Gly content in leaves, phosphorylated B_6_ vitamer content in unpolished seeds, PN-Gly content in polished seeds, leaf length under normal conditions, as well as *AtPDX1.1* and *AtPDX2* expression values in leaves displayed moderate deviation from normality and homoscedasticity, possibly associated with the low sample size.

The vitamin B_6_ contents assessed by the yeast bioassay, the metabolite profiles, the phenotypic characteristic and the *Xanthomonas oryzae* lesion lengths in transgenic lines were compared to the controls using a Student’s *t*-test (α = 0.05). A Bilateral test was applied and type 2 (homoscedasticity) or 3 (hetereoscedasticity) was determined by the Fischer’s test of equality of variance (F-test, α = 0.05).
