# Supplementary material for: Enhancement of vitamin B6 levels in rice expressing Arabidopsis vitamin B6 biosynthesis de novo genes
Source: Plant J. 2019 Jul 11;99(6):1047–65. doi: 10.1111/tpj.14379 (PMC6852651; doi:10.1111/tpj.14379)
Supplement: Supplementary file 4 [file TPJ-99-1047-s004.docx]

**Figure S1. Molecular characterization of the generated dual-expressing *AtPDX1.1* and *AtPDX2* transgenic rice lines in the T_0_ generation.**

Transformation 1, 2 and 3 refer to the three independent transformations performed to generate transgenic rice lines having constitutive expression (called *35S* lines). A small pool of endosperm-specific expression lines (called *Glob* lines) were also generated. The construct used for the latter lines was the same as that shown in **Figure 2a**, with the exception that the *CaMV 35S* promoter was replaced with the *globulin* promoter. **(a)** Identification of putative transformants by PCR for *AtPDX1.1* and *AtPDX2* transgenes. **(b)** Determination of T-DNA integration and copy number by Southern blot, employing a probe against the *hptII* gene.

**Figure S2. Analysis of total vitamin B_6_ contents of transgenic rice lines in the T_1_ generation.**

Vitamin B_6_ quantification by a yeast bioassay in leaves of 45-day-old plants. **(a)** Vitamin B_6_ content in undiluted leaf sample extracts. Average ± S.D. of 3 biological replicates (except for *35S*-7a (n = 2) and *35S*-7b (n = 1)). Standard deviation is not indicated for samples with at least one O.D. (optical density) value above the linear range of the standard curve indicated by the area above the dotted line. **(b)** Vitamin B_6_ content in 30-fold diluted leaf sample extracts. Average ± S.D. of 3 biological replicates (except for *35S*-7a (n = 2) and *35S*-7b (n = 1)). nd: not detected.

**Figure S3. Assignment of a glucosylated B_6_ vitamer in rice leaf extracts.**

**(a)** Elution profiles of leaf extracts before (blue) and after (red) β-glucosidase treatment using the transgenic control line p1300. To facilitate the visualization, untreated and treated sample profiles were offset by 500 and 1400 respectively, with respect to the baseline of the standards. See **Figure 5** for numbering scheme of peaks 1-6. **(b)** Elution profiles of the individual peaks 1/2 or 3 before and after β-glucosidase treatment as indicated. In each case, 100 μL of the collected peak(s) was incubated ± β-glucosidase (10 μL of 15 mg.mL^-1^ stock) for 2 h at 37°C, followed by heating to 99°C for 3 min, and 50 μL re-injected for chromatography. To facilitate the visualization, chromatograms of the collected peaks were offset by 100 unit increases relative to the baseline of the standards. The difference in retention times between part **(a)** and **(b)** are likely due to small changes in pH.

**Figure S4. Rice *PDX* gene expression patterns across different tissues.**

Data were computationally generated using Genevestigator (Hruz et al., 2008).

**Figure S5. *PDX* transgene expression, protein accumulation and vitamin B_6_ content, in the rice *Glob* lines**

**(a)** *AtPDX1.1* and **(b)** *AtPDX2* transcript expression levels in *Glob* transgenic rice lines (T_2_ generation) compared to wild-type (TP309) and the empty vector control (p1300). Average ± S.D. of 3 biological replicates. Tukey's multiple comparison test (*p* < 0.05) for each tissue. Western blot analysis of **(c)** AtPDX1.1 and **(d)** AtPDX2 protein abundance (upper panel) and Ponceau staining of the nitrocellulose membrane (lower panel) in control and transgenic rice lines. Fifty micrograms of total rice proteins and 30 μg of total Arabidopsis leaf proteins were probed with peptide antibodies specific to AtPDX1.1 (Raschke et al., 2011) or AtPDX2 (Tambasco-Studart et al., 2007). M: protein molecular-weight marker; L: leaves; US: unpolished seeds; PS: polished seeds; At: *Arabidopsis thaliana*. **(a-h)** Vitamin B_6_ measurements by yeast bioassay, *PDX* transgene expression levels and immunochemical analyses of the *Glob* lines presented in **Figure S5** were performed simultaneously to the analysis of the *35S* lines presented in **Figures 2 and 7**. **(e)** Vitamin B_6_ content in fresh mature unpolished and polished seeds and in the embryo from plants in the T_2_ generation grown in the greenhouse. Average ± S.D. of 4 biological replicates. Student's *t*-test (p1300 *vs* transgenic lines), * *p* < 0.05, ** *p* < 0.01. **(f)** Vitamin B_6_ content in dry mature unpolished and polished seeds of the samples as in **(e)**. **(g)** Vitamin B_6_ content in leaf sample extracts of *Glob* lines in the T_2_ generation compared to wild-type (TP309) and empty vector control (p1300). Average ± S.D. of 4 biological replicates. Student's *t* test (p1300 *vs* transgenic lines and TP309), * *p* < 0.05, ** *p* < 0.01. **(h)** Vitamin B_6_ content in root sample extracts from mature plants in the T_2_ generation grown under greenhouse conditions. Average ± S.D. of 4 biological replicates. Student's *t*-test (p1300 *vs* transgenic lines), * *p* < 0.05, ** *p* < 0.01. Values above the bars represent the fold increase compared to p1300.

**Table S1. Primers used for the molecular characterization of generated transgenic rice lines.**

**Table S2. Primers used for real-time quantitative PCR analysi*s.***
